# Dataset on Weather-related Disasters in Agriculture (WDA) in Italy 2005–2021

**DOI:** 10.1016/j.dib.2025.111323

**Published:** 2025-01-22

**Authors:** Antonella Pontrandolfi, Roberta Alilla, Flora De Natale, Roberto Nuti, Barbara Parisse, Antonio Gerardo Pepe

**Affiliations:** Council for Agricultural Research and Economics – CREA, Via della Navicella 2/4 00184 Rome, Italy

**Keywords:** Risk assessment, Weather extremes, Agriculture, Climate change

## Abstract

The geodatabase on weather-related disasters in agriculture is a part of the cloud storage which hosts the materials of the Observatory for Agricultural meteorology and climatology of the Council for Agricultural Research and Economics (CREA). A specific relational SQL geodatabase has been created by data entry from the official decrees of disaster declaration of the Italian Ministry of Agriculture, food sovereignty and forests within the “National Solidarity Fund for natural disasters in agriculture” (Italian law d.lgs. 102/2004). From this database, managed by CREA for internal research purposes, a dataset has been extracted for the period from 2005 to 2021 and published in the Zenodo repository in text format (.csv file) annotated with discovery and structural metadata. The dataset aims to make available useful data for weather-related risk assessment in agriculture and for agrometeorological analyses of extreme weather events leading to disasters. The following types of weather-related disasters in agriculture are included in the dataset: drought, excess of snow, frost, hail, heat stress, heavy rain leading to flood, persistent rain, tornado, strong wind and sirocco wind. The dataset will be periodically updated.

Specifications Table

**Table:** 

Subject	Agricultural Sciences; Environmental Sciences (Management, Monitoring, Policy and Law)
Specific subject area	Weather-related disasters in agriculture officially declared within the National Solidarity Fund for natural disasters in agriculture in Italy
Type of data	Table Processed
Data collection	The dataset is extracted from the CREA geodatabase “**Weather-related disasters in agriculture**”. The decrees of disaster declaration of the Italian Ministry of Agriculture, food sovereignty and forests within the “National Solidarity Fund for natural disasters in agriculture” were analysed and the main data have been imported in a specific relational SQL geodatabase, assigning the official related administrative units NUTS 2, 3 and municipalities. The codes and names of the official administrative units were obtained from the Italian National Institute of Statistics (Istat), updated to 2016. The dataset covers the Italian area and will be periodically updated*.*
Data source location	Country: Italy Institution: Council of Agricultural Research and Economics – CREA; Research Centre for Agriculture and Environment, Rome Italy (website: https://agrometeo.crea.gov.it/ Observatory for Agricultural meteorology and climatology)*.*
Data accessibility	Repository name: Zenodo Data identification number: 10.5281/zenodo.14499762 Direct URL to data: https://doi.org/10.5281/zenodo.14499762 Instructions for accessing these data: None
Related research article	*None.*

## 1 Value of the Data

• The data on officially declared weather-related disasters in agriculture (WDA) are important from a scientific point of view for better understanding the impacts on agriculture of extreme weather events in different time and spatial scales
• The data can also be useful in the analysis of the “vulnerability” in climatic risk studies (as a direct or proxy variables)
• At a decision-making level, the risk management policy design can benefit from analysis using these data, improving robustness and effectiveness
• The data can be used for further developments in risk studies matching the extreme weather events (hazards) and their impacts (disasters), also testing the ability of different agrometeorological indices (hazard indices) to better catch the potentially damaging weather extremes in different spatial and time scales

## 2 Background

Agriculture is highly dependent on climate and weather conditions and extreme weather events leading to disasters affecting agricultural productions and farms’ incomes, therefore in Italy huge public investments are dedicated to risk management policies in agriculture.

In the context of climate change, the extreme weather events are expected to rise the occurrence of “disasters”, defined by the Intergovernmental Panel on Climate Change as serious alterations and socio-economic disruptions [1].

Some studies pointed out the importance of analysing the official disaster declarations in order to improve the risk policy design [2,3], also because similar extreme weather events can have different impacts depending on the vulnerability of the exposed systems [4]. Other studies investigated the relation between a specific type of extreme weather event and impacts in agriculture [5].

Specific studies referring to Italy have been carried out on the distribution in time and space of WDA [6] and on the link between WDA and agrometeorological indices [7,8].

In the Italian risk management scheme for agriculture, a WDA is recognized when damages are caused by a specific extreme weather event. The damages are certified in official disaster declarations of the Italian Ministry of Agriculture, food sovereignty and forests (Masaf). Since the ‘70 s, Italy has a “National Solidarity Fund for natural disasters in agriculture”, reformed in 2004 (legislative decree n. 102/2004) [9]. According to the law, a WDA is caused by an extreme weather event (such as frost, storms and hail, heavy rain or drought, etc.) damaging more than 30 % of the average annual production. In addition, damages on farms’ structures (stables, greenhouses, sheds, etc.) and infrastructures connected to agricultural activities (mostly collective drainage and irrigation channels, rural roads, etc.) are considered, in terms of restoration costs or the economic value of the structure before the damage. After the occurrence of the event, the involved administrative Region produces a request for a disaster declaration, supported by technical reports that describe the event, the location and the damages occurred. The WDA declaration decree is issued by the Masaf if the regional request meets all the law requirements. The declaration reports the type of extreme weather event leading to disaster, the date of its occurrence, the kind of damages caused to agriculture, and the territorial administrative unit involved (provinces – NUTS 3 or municipalities).

## 3 Data Description

The WDA dataset is composed by 1159 records with 11 attributes [10]. Each record corresponds to a WDA declared in a specific time (date) and space (one administrative unit - NUTS 3 – of the 107 Italian ones) [11]; therefore, each record is identified by a unique code given by the association date-province, as shown in Table 1, which reports a description of the dataset attributes. Table 2 shows the code list applied for the attribute “Extreme weather event leading to disaster” and the corresponding definitions adopted by the Italian law scheme [12]. All this information is part of the Structural metadata published in the Zenodo repository.

**Table 1:** Attribute description.

Attribute	Description	Codelist	Type	Format
ID	Record identification code		string	yyyy-CodProv-DOY[start]-DOY[end] where yyyy: year of occurrence DOY: day of the year (1–366)
CodReg	The official ISTAT code for Regions NUTS 2	https://www.iso.org/obp/ui/#iso:code:3166:IT	string	
CodProv	The official ISTAT code for Provinces NUTS 3	https://www.iso.org/obp/ui/#iso:code:3166:IT	string	
Den_Prov	The official name of Provinces	https://www.iso.org/obp/ui/#iso:code:3166:IT	string	
Year	The year of occurence		string	yyyy
Extreme weather event leading to disaster	The type of weather event leading to disaster	Weather event codelist	string	
Start date	Start date of the disaster		date	yyyy-mm-dd
End date	End date of the disaster		date	yyyy-mm-dd
Damages on productions	Presence or not of damages on productions		boolean	*Y*=True or N=False
Damages on farm structures	Presence or not of damages on farm structures		boolean	*Y*=True or N=False
Damages on infrastructures	Presence or not of damages on infrastructures		boolean	*Y*=True or N=False

**Table 2:** “Extreme weather event leading to disaster” attribute: list of values and related definitions.

Value	Definition
Hail	Frozen water in the atmosphere that falls in the form of ice lumps of varying sizes; (Ministry decree n. 9,402,305 of 2020/12/29)
Frost	Temperature below 0 °C due to the presence of cold air masses. The negative effects for violence and intensity of the weather event must be found on neighbouring areas and/or crops; (Ministry decree n. 9,402,305 of 2020/12/29)
Drought	Extraordinary rainfall deficit compared to the normal value of the period, which causes a soil water content below the critical humidity limit and/or depletion of water sources to the point of making impossible the emergency irrigation interventions. The event must have significant effects on the vitality of the plants and the effects must be found on neighbouring areas and/or crops; (Ministry decree n. 9,402,305 of 2020/12/29)
Heat stress	Temperature higher than normal, which, due to its duration and/or intensity, causes significant effects on the physiology of plants, with consequent loss of production. The negative effects for violence and intensity of the weather event must be found on neighbouring areas and/or crops.
Heavy rain leading to flood	Natural disaster in the form of flooding, due to exceptional precipitation events, of natural and/or artificial watercourses and waterbodies invading the surrounding areas and are accompanied by the transport and deposit of solid and incoherent material. The negative effects for violence and intensity of the weather event must be found on neighbouring areas and/or crops or near areas with similar orographic characteristics; (Ministry decree n. 9,402,305 of 2020/12/29)
Persistent rain	Excess of water availability in the soil and/or of rainfall exceeding the average for the period, causing damages to productions. The negative effects for violence and intensity of the weather event must be found on neighbouring areas and/or crops or near areas with similar orographic characteristics; (Ministry decree n. 9,402,305 of 2020/12/29)
Tornado	A tornado is a violent rotating column of air that reaches to the ground from a storm cloud in the shape of a condensation funnel created and maintained by strong inflowing winds.
Strong wind	Wind reaching at least the 7th degree of the Beaufort scale, with direct mechanical/physical damages on productions and structures. The negative effects for violence and intensity of the weather event must be found on neighbouring areas and/or crops or near areas with similar orographic characteristics; (Ministry decree n. 9,402,305 of 2020/12/29)
Siroco wind	More or less regular or violent wind from South-East combined with air temperature of at least 30 °C, which causes damages on productions due to its duration and/or intensity. The negative effects for violence and intensity of the weather event must be found on neighbouring areas and/or crops; (Ministry decree n. 9,402,305 of 2020/12/29)
Excess of snow	Snow precipitation which causes significant mechanical/physical damages on productions and structures due to its duration and/or intensity. The negative effects for violence and intensity of the weather event must be found on neighbouring areas and/or crops; (Ministry decree n. 9,402,305 of 2020/12/29)

**Fig. 1:**
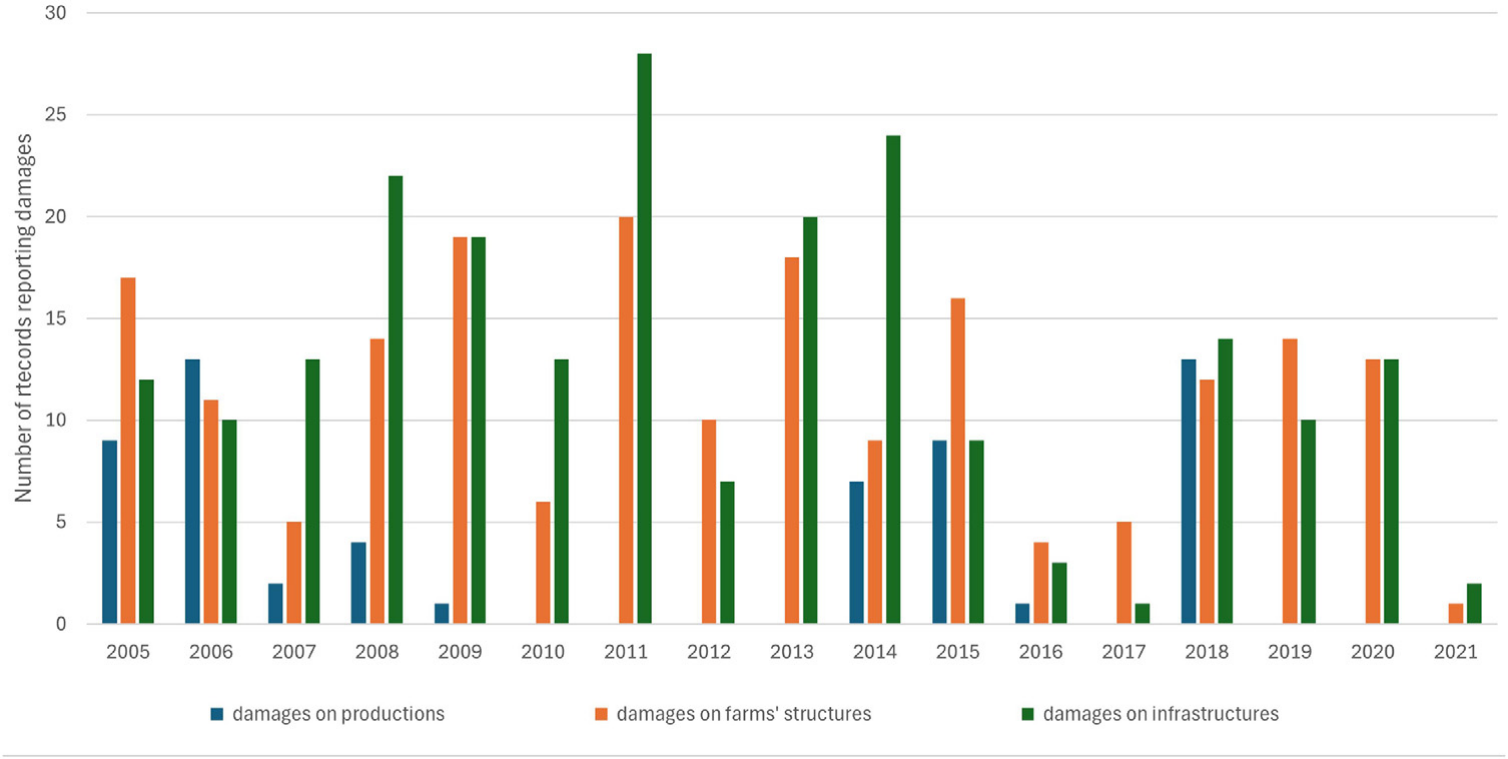
Number of records per year for heavy rain leading to flood.

**Fig. 2:**
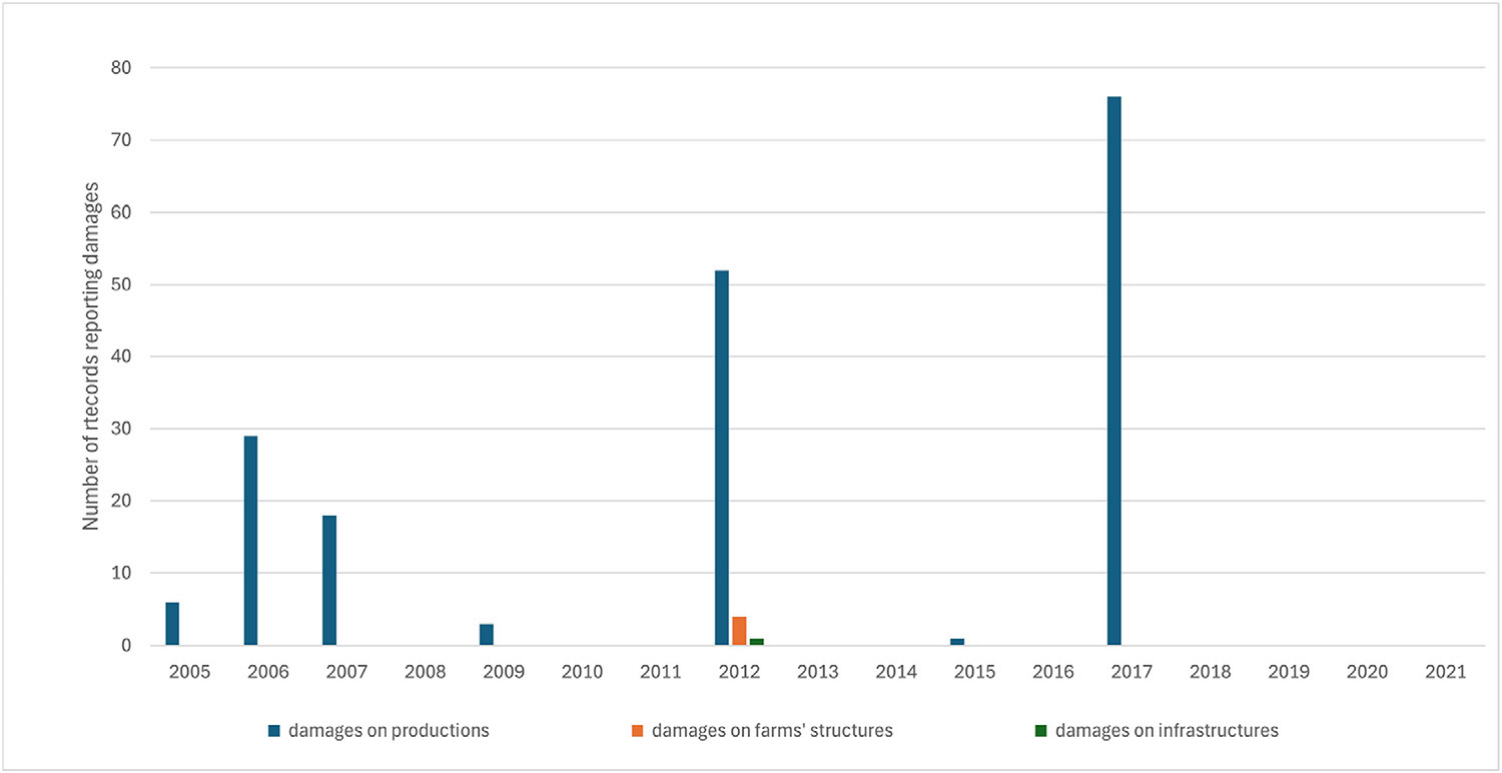
Number of records per year for drought.

Table 3 summarizes the WDA dataset content, in terms of the total number of records per type of extreme event leading to disaster, and per kind of damage caused, over the period 2005–2021. Figs. 1 and 2 show the distribution over the years for the two most frequent types of WDA (i.e. heavy rain leading to floods and drought). It should be noticed that a WDA can imply multiple kinds of damage (e.g. on both production and farms’ structures).

**Table 3:** Number of records per type of extreme weather events leading to disaster and per kind of damages.

“Extreme weather event leading to disaster”	No. of records reporting damages on productions	No. of records reporting damages on farms' structures	No. of records reporting damages on infrastructures
Drought	185	4	1
Excess of snow	17	54	14
Frost	158	15	2
Hail	18	62	
Hail and tornado	1		
Heat stress	11		
Heavy rain leading to flood	59	194	220
Persistent rain	14	53	80
Sirocco wind	5		
Strong wind	12	59	3
Strong wind and heavy rain leading to flood	7	6	2
Strong wind, hail and heavy rain leading to flood	1		
Tornado	14	110	5

## 4 Experimental Design, Materials and Methods

The data derive from the analysis and acquisition of the information of the decrees of WDA declaration issued over the years by the Italian Ministries of Agriculture, precisely the type of extreme weather event leading to disaster, the date of its occurrence, the kind of damages, and the territorial administrative unit involved (provinces – NUTS 3 or municipalities).

**Fig. 3:**
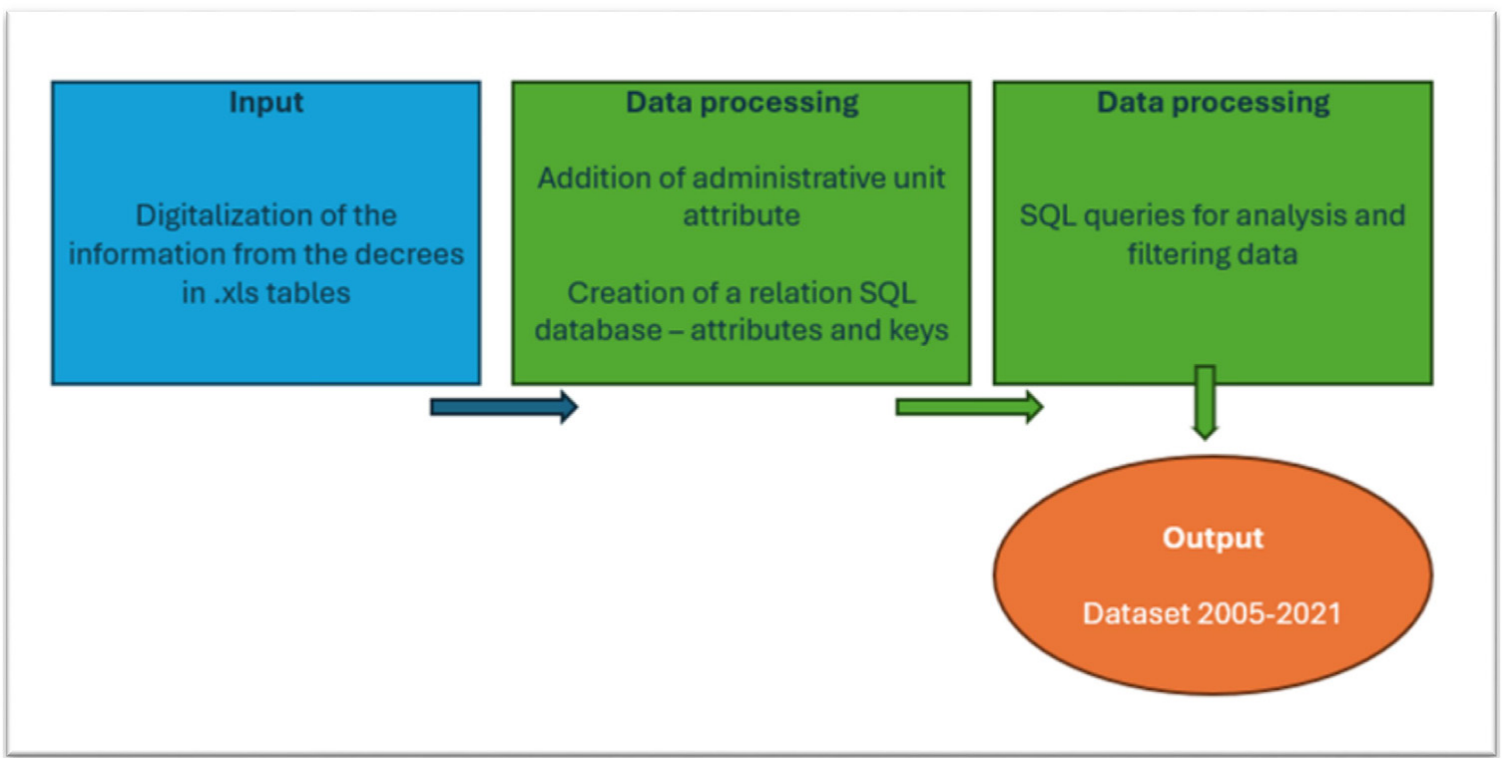
Data processing.

Collected data have been organized in tables. The official Istat codes of administrative units of the areas affected (NUTS 2, 3 and municipalities, updated to 2016) been added to each record [13] (Fig. 3). Particular attention has been given to this step, because of the institutional reforms of the administrative units’ boundaries implemented over the past years (these data are stabilized at 2016). In case of changes in administrative boundaries, the correct code assignment was made following an in-depth analysis of the decree documentation.

**Fig. 4:**
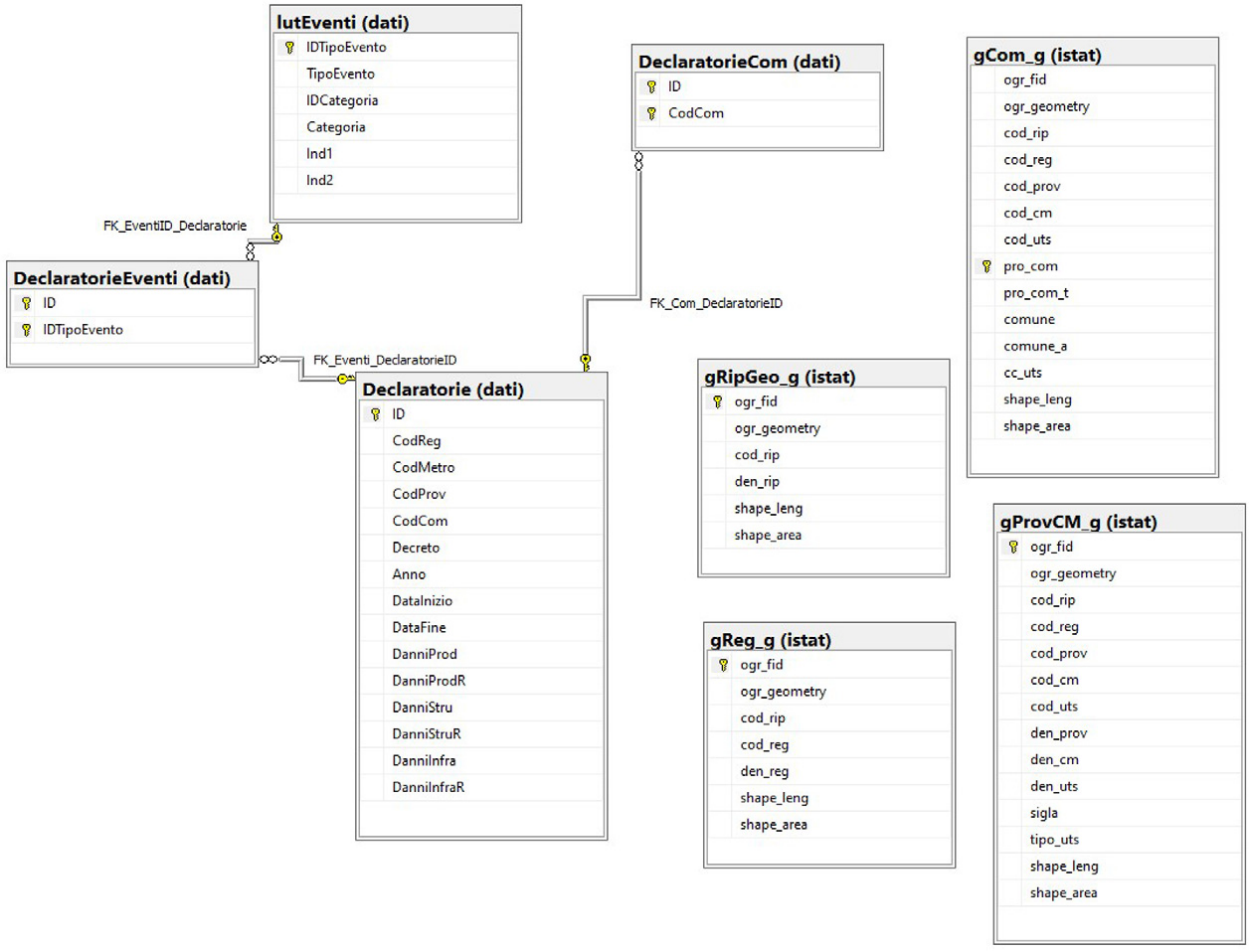
The dataset source: the schema of the relational SQL geodatabase.

In a second step, the data have been structured in a dedicated relational SQL geodatabase, which is yearly updated. The database is composed by several tables organized following the ER Model (Entity-Relationship) represented in Fig. 4, which visually represents the relationships between the tables. The main relations refer to:

- the table “Declaratorie” (declarations), which has a one-to-many relation with the table “DeclaratorieEventi” (Extreme weather events codes)
- the table “DeclaratorieEventi” is linked to the table “lutEventi” (type of extreme weather event) through the event code (“IDTipoEvento”)
- the table “Declaratorie” has a one-to-many relation with the table “DeclaratorieCom” (administrative units) as one declaration can contain multiple provinces/municipalities affected by the extreme weather event

Additional tables provide further attributes derived from Istat generalized GIS territorial layers (gCom-g, gReg_g, and gprovCM_g - ISTAT).

The data can be aggregated at different levels through SQL queries (by area, provinces, territorial unit, and type of events, in different periods).

The WDA **dataset** has been extracted by SQL queries from the geodatabase, filtering the period from 2005 (post-reform) to 2021 (last stabilized update) at NUTS 3 level .

**Fig. 5:**
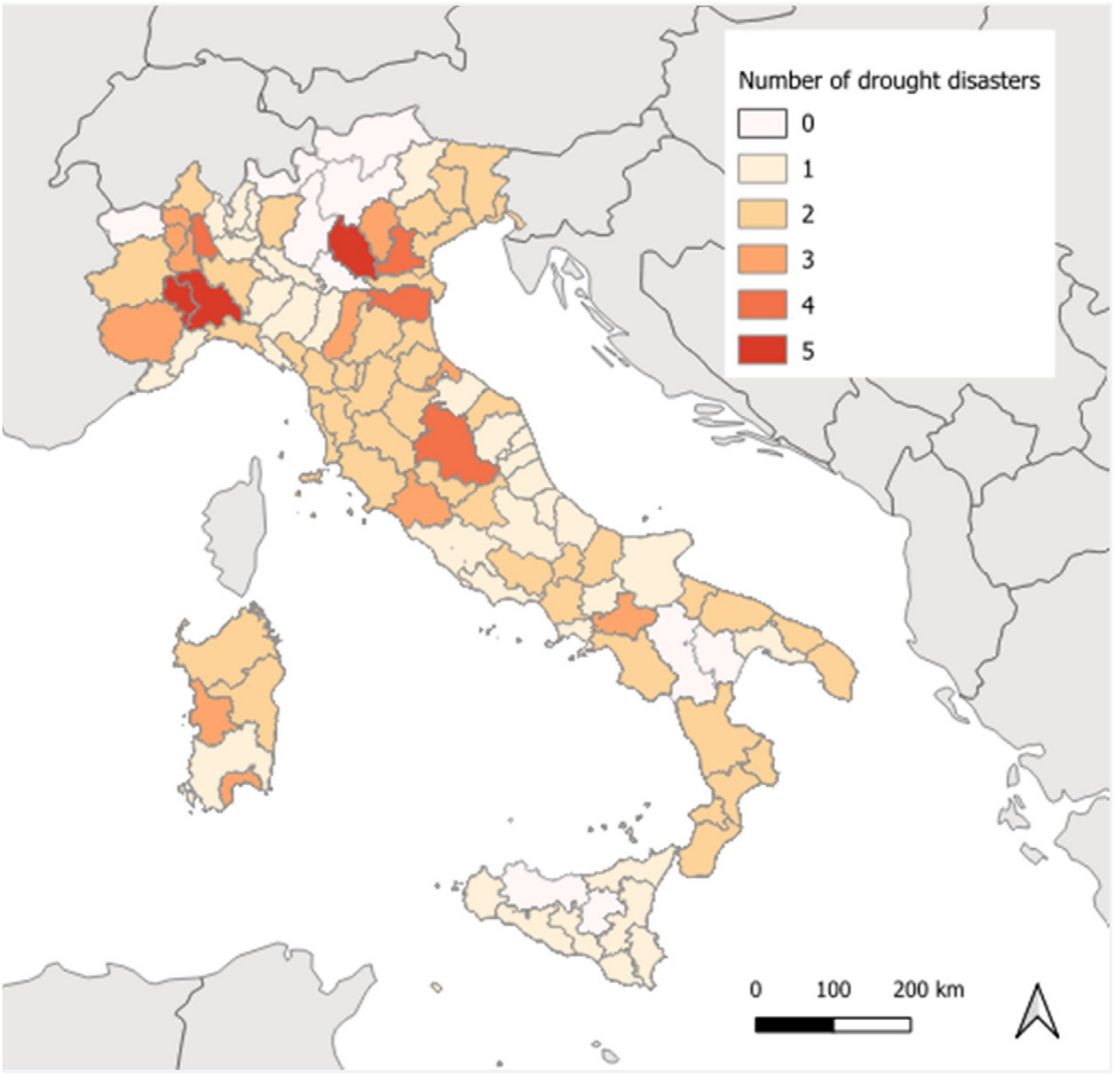
Number of drought disasters declared per Province in the period 2005–2021.

The Istat codes allow the data to be georeferenced by performing a table join between them and the polygon layer of the NUTS 3 administrative units, that can be downloaded from the Istat official website [13]. In this way, in addition to territorial statistics, the data can also be useful for some geographical analyses. As an example, Fig. 5 shows a spatial analysis about the occurrence of drought disasters in Italy.

Finally, data are annotated with Discovery (global attributes, which describe the whole dataset) and Structural (variable specific attributes) metadata, available in the Zenodo repository, which meet the domain relevant community standards, as required by the FAIR principles [14]. Discovery metadata are compliant with EN ISO 19,115 and EN ISO 19,119 INSPIRE standards [15]*.*

## Limitations

In some cases, the attribution of the type of extreme weather event leading to disaster reported in the disaster declarations showed some ambiguities, therefore it required a more in-depth analysis before data entry. For instance, some disasters caused by “persistent rain” occurred in a very short period (few days), while some others caused by “heavy rain leading to flood” occurred in a very prolonged period (many months). Anyway, these uncertainties are very few and, in our opinion, do not remarkably influence analyses of these data. Another dataset limitation deriving from the decrees is the lack of information about the kind of crops affected by damages on production, which could really improve the risk assessment in agriculture. Finally, it is important to underline that the areal units of analysis could change in case of future changes in administrative units’ boundaries due to institutional reforms, therefore an additional effort could be required in updating the dataset for matching the future WDA with their right Istat codes.

## Ethics Statement

This work does not involve human subjects, animal experiments, or any data collected from social media platforms. The authors have read and follow the ethical requirements for publication in Data in Brief

## Credit Author Statement

Antonella Pontrandolfi: Conceptualization, Methodology; Antonio Gerardo Pepe: Data and geodatabase curation; Roberto Nuti: Data curation; Antonella Pontrandolfi: Original draft preparation; Antonella Pontrandolfi, Alilla Roberta, Flora De Natale and Barbara Parisse: Writing- Reviewing and Editing; Alilla Roberta, Flora De Natale and Barbara Parisse: Metadata curation.

## Data Availability

ZenodoDataset on Weather-related disasters in agriculture in Italy - WDA (Original data).
